# Effect of strontium-doped bioactive glass-ceramic containing toothpaste on prevention of artificial dentine caries formation: an in vitro study

**DOI:** 10.1186/s12903-022-02321-z

**Published:** 2022-07-16

**Authors:** Lin Lu Dai, May Lei Mei, Chun Hung Chu, Edward Chin Man Lo

**Affiliations:** 1 Department of General Dentistry, Shanghai Ninth People’s Hospital, Shanghai Jiao Tong University School of Medicine, Shanghai, China; 2 College of Stomatology, Shanghai Jiao Tong University, Shanghai, China; 3 National Center for Stomatology, Shanghai, China; 4 National Clinical Research Center for Oral Diseases, Shanghai, China; 5 Shanghai Key Laboratory of Stomatology, Shanghai, China; 6 Faculty of Dentistry, The University of Hong Kong, 34 Hospital Road, Sai Ying Pun, Hong Kong SAR, China; 7 Faculty of Dentistry, University of Otago, Dunedin, New Zealand

**Keywords:** Bioglass, Prevention, Strontium, Decay

## Abstract

**Backgroud:**

Root caries in aging population was prevalent worldwide. Due to the absence of enamel and specific structure of dentine, bacteria are able to penetrate further into dentine at an earlier stage of lesion development. The aim of this study was to investigate the effect of adding of a strontium-doped bioactive glass-ceramic (HX-BGC) to a fluoride-free toothpaste on prevention of formation of artificial dentine caries.

**Methods:**

Thirty-six human tooth specimens were allocated to three groups (*n* = 12 per group). Group 1 treated with slurry containing a fluoride-free toothpaste and 5% HX-BGC, Group 2 was treated with fluoride-free toothpaste slurry, and Group 3 received deionized water as a negative control. The specimens were subjected to four cycles (15 h demineralization and 8 h remineralization for one cycle) of biochemical cycling. A mixed suspension of five bacteria species (*Streptococcus mutans*, *Streptococcus sobrinus*, *Lactobacillus acidophilus*, *Lactobacillus rhamnosus*, and *Actinomyces naeslundii*) were prepared in brain heart infusion broth with 5% sucrose and used as acidic challenge in biochemical cycling. Subsequently, surface morphology of the dentine lesion was assessed by scanning electron microscopy, while the lesion depths and mineral loss were assessed by micro-computed tomography.

**Results:**

The mean lesion depths in dentine in Groups 1 to 3 were 87.79 ± 16.99 μm, 101.06 ± 10.04 μm and 113.60 ± 16.36 μm, respectively (*p* = 0.002). The mean amounts of mineral loss in Groups 1 to 3 were 0.82 ± 0.10 g/cm^3^, 0.89 ± 0.09 g/cm^3^ and 0.96 ± 0.11 g/cm^3^, respectively (*p* = 0.016). No obvious differences in the surface morphology were seen among the groups.

**Conclusion:**

Addition of strontium-doped bioactive glass-ceramic to fluoride-free toothpaste has potential to reduce formation of dentine lesions.

## Background

Dental caries remains the most common chronic disease worldwide [1]. Root caries is predicted to rise due to a growth in aging population, that is associated with increased gum recession and extended exposure of tooth roots to a carious environment [2]. In root caries, bacteria are able to penetrate further into dentine at an earlier stage of lesion development due to the absence of enamel and due to the porous structure of dentine. Dentine is composed of 70% mineral and 20% organic matter (with 10% water), and collagen type I acts as a scaffold for mineral deposition [3]. Dramatic effects occur during the progress of caries, including collapse of collagen due to dissolution of inorganic minerals and the formation of a cavity [4]. Current concepts on restoring root caries focus on bioactive materials which can promote remineralization [5]. Topical fluoride application has been used as a non-invasive approach to arrest and prevent caries. Apart from fluoride agents, new anti-caries agents with potential bioactivity and biocompatibility have also been introduced to manage caries [6].

A new type of bioactive glass-ceramic, named Huaxi bioactive glass-ceramic (HX-BGC, consists of SiO_2_, P_2_O_5_, Na_2_O and SrO) which contains strontium has recently been invented by Sichuan University and used in treating dental hypersensitivity [7]. With the addition of strontium, HX-BGC can suppress acid production and has potential inhibitory effect on the growth of cariogenic bacteria [8]. Besides, HX-BGC has a preventive effect on the formation of artificial carious lesions on sound enamel and dentine [9]. Furthermore, our previous study showed that HX-BGC can reduce mineral loss and has remineralizing potential on demineralized dental tissues [10]. Most of toothpastes in the market contain fluoride or other anti-caries agents, but very few toothpastes contain bioactive glasses which have a potential effect on caries management. The aim of this study was to investigate the effect of adding HX-BGC to a fluoride-free toothpaste on preventive effect of artificial dentine caries formation.

## Materials and methods

Ethical approval (UW 21-375) for use of extracted human teeth was obtained from the University of Hong Kong in May, 2021.

### Preparation of specimens and bacterial suspensions

Twelve extracted human molars were selected from the collection of extracted teeth of a dental teaching hospital. These teeth were stored in a 0.5% thymol solution before use. The preparation of tooth specimens followed a standard protocol used in a previous study [10]. The selected molars were cut by a low-speed cutting machine (ISOMET 1000, Buehler, LakeBluff, IL, USA) under running deionized water to produce 36 dentine slices (3 slices from each tooth) with a thickness of 2 mm. Subsequently, each dentine slice was cut evenly into four specimens. The surfaces of the specimens were polished using micro-fine 4000 grid sand paper and washed in deionized water. Then, a stereomicroscope was used to examine the specimens so as to exclude specimens with cracks or defects. The surface of each specimen was covered by an acid-resistant nail varnish, except for half of the upper surface. Then the specimens were stored in deionized water at 4 °C. Afterwards, the specimens were autoclaved at 120 °C before use [11]. A pilot study on the pH changes in the bacterial suspensions over 72 h was conducted before we used the mixture of suspensions as a demineralizing agent during the biochemical cycling. Five species of cariogenic bacteria were used in this study and they were *Streptococcus mutans* (*S. mutans*, ATCC 35,668), *Streptococcus sobrinus* (*S. sobrinus*, ATCC 33,478), *Lactobacillus acidophilus* (*L. acidophilus*, ATCC 9224), *Lactobacillus rhamnosus* (*L. rhamnosus*, ATCC 10,863), and *Actinomyces naeslundii* (*A. naeslundii*, ATCC 12,104). Five bacterial suspensions, each containing one bacterial species, were prepared in a brain heart infusion (BHI) broth with 5% sucrose to a cell density of 10^8^ cells/mL. Subsequently, the five bacterial suspensions were mixed and incubated in an anaerobic chamber (Forma Anaerobic Chamber; Thermo Fisher Scientific Inc., MA, USA) for 8 h before use in the cycling.

### Biochemical cycling and experimental treatment

**Fig. 1:**
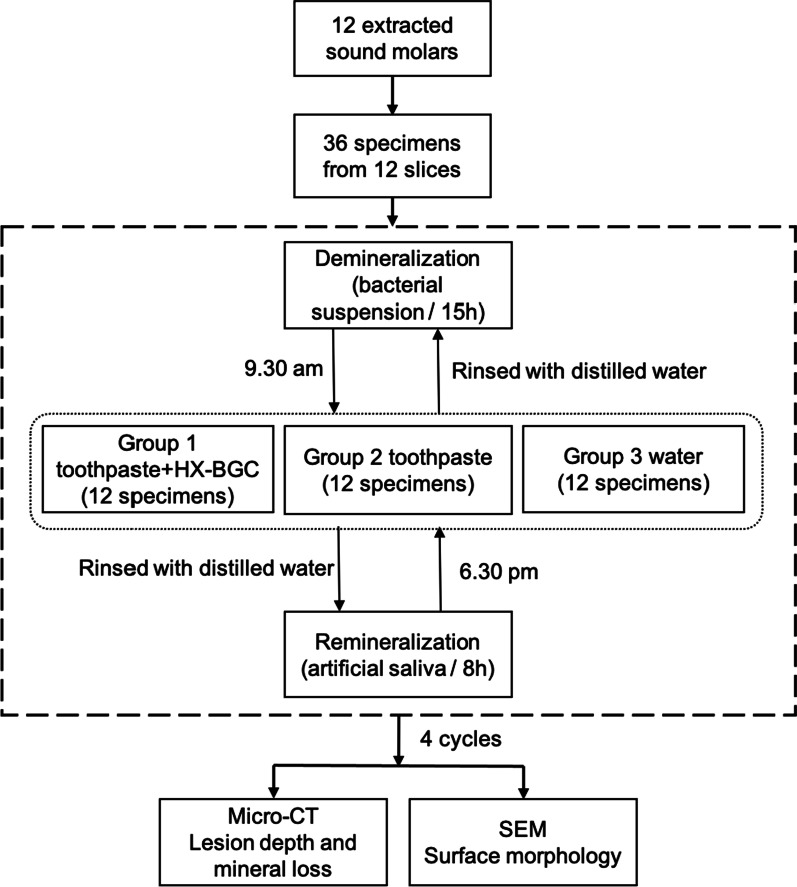
The flowchart of the experimental procedure using the biochemical model

A fluoride-free toothpaste (NUK, Germany) was used in this study. The main components of this toothpaste were hydrated silica, propylene glycol, xylitol, sodium C14-16 olefin sulfonate, calcium citrate, tocopheryl acetate and sodium methylparaben. The HX-BGC powder was provided by Dencare (Chongqing) Oral Care Co., Ltd. The main components were as follows: SiO_2_ (12–45%), P_2_O_5_ (10–35%), CaO_2_ (5–48%), Na_2_O (5–15%), SrO (3.5–4.9%) and F (1.5–2.1%). The particle size of HX-BGC was ranged from 23.5 to 40.9 μm [8]. The concentration of HX-BGC was 5 wt% [12]. Novamin was used as a reference to determine the ratio of the bioactive glass to the slurry. The reason we used slurry is to mimic when the toothpaste was diluted by the water/saliva. However, it dose make a difference from 5 wt% toothpaste. Three prepared specimens from each tooth slice were randomly allocated into one of three intervention groups so that each group had 12 specimens. The fluoride-free toothpaste slurry was prepared by adding the toothpaste into water at a ratio of 1:3. The toothpaste slurry with HX-BGC was prepared by adding HX-BGC at a concentration of 5% into the fluoride-free toothpaste slurry. However, it does make a difference from 5 wt% toothpaste. The toothpaste slurries were prepared just before the cycling and autoclaved for 30 min before use. A biochemical cycling model was modified and used in this study [13]. All the specimens were subjected to a bacterial suspension (5 species cariogenic bacteria) for 15 h in an anaerobical environment at 37 °C. The bacterial suspensions were prepared freshly before every cycle. After the 15 h demineralization, the specimens were rinsed with deionized water and were applied with the corresponding agent according to their group assignment described. The tooth specimen surfaces without nail polish were treated with the corresponding suspensions for 2 min using a micro-brush (Premium Plus Internatinal Ltd., Hong Kong) according to its group allocation [10]. The tooth specimens in Group 1 were placed in the fluoride-free toothpaste with 5% HX-BGC slurry for 2 min. The tooth specimens in Group 2 were placed in the fluoride-free toothpaste slurry for 2 min. The specimens in Group 3 were placed in deionized water as a negative control. Subsequently, the specimens were rinsed with deionized water to remove the applied agents. This prepararion process took 30 min to complete for all 36 specimens. The specimens then went through an 8 h immersion in artificial saliva (20 mM 4-(2-hydroxyethyl)-1-piperazineethanesulfonic acid; 1.5 mM calcium chloride; 0.9 mM potassium dihydrogen phosphate and 150 mM potassium chloride, pH = 7) at 37 °C. The demineralization (without artificial saliva) and remineralization (placed in artificial saliva) cycles was repeated for 4 days. The cycles were done in triplicate. The flow of procedures is shown in Fig. 1.

### Lesion depth and mineral loss

The specimens were collected after the biochemical cycling. Each specimen was scanned by micro-computed tomography (Micro-CT) (SkyScan1172; SkyScan, Antwerp, Belgium). The voltage and current settings were 80 kV and 100 μA, respectively. The image pixel size was set at 7.95 μm and the X-rays were cut-off by a 0.5 mm aluminium filter. Scanning time was approximately 125 min for each specimen. Ten specimens from each group were scanned and reconstructed by using the NRecon reconstruction software (SkyScan, Antwerp, Belgium). The reconstructed three-dimensional images were analyzed by a CT data analyzing software (CTAn, SkyScan, Antwerp, Belgium). Ten reconstructed cross-sectional images from each specimen were randomly selected for taking measurements of the lesion depth and mineral loss (Δ mineral density, g/cm^3^). For measuring lesion depth, there was an internal control reference line, which was a virtual line formed by extending from the non-demineralized area of specimen surface to the artificial lesion [13]. To measure mineral loss, two mineral phantoms with the mineral density values of 0.25 g/cm^3^ and 0.75 g/cm^3^ were used for calibration. The mineral loss was calculated by subtracting the mineral density value of demineralized area from the sound area of the corresponding dental tissue [14].

### Morphology of dentine surface

Two specimens from each group were ultrasonically washed three times with deionized water to remove the biofilm and toothpaste slurry. Then, they were fixed in 2.5% glutaraldehyde overnight, followed by washing them using deionized water. Subsequently, the specimens were dehydrated in a series of ethanol solutions with different concentrations (75%, 80%, 95%, 100%). The dehydrated specimens were further dried in a critical-point dryer (Leica EM CPD300, Germany) and sputter-coated with carbon. After the above-mentioned steps, surface of the specimens was observed under a scanning electron microscope (SEM, Hitachi S-4800FEG Scanning Electron Microscope, Hitachi Ltd., Tokyo, Japan). The morphology change in dentine was purely for descriptive purpose.

### Statistical analysis

Data on the lesion depths and mineral loss (Δ mineral density value) of dentine in the three study groups were analyzed by using the statistics software IBM SPSS version 27. Data collected from the CTAn were first assessed for normality using the Shapiro–Wilk test and then the differences in lesion depth and mineral loss among groups were tested by using one-way analysis of variance (ANOVA). The significance level of all statistical tests was set at 5%.

## Results

In the pilot study, the pH changes of the bacterial suspension at different time points over 72 h were monitored. The pH of the suspension mixture was 7.0 during the first 6 h, then it decreased to around 5.5 at 8 h. The pH further dropped to 4.0 at 24 h and remained steady during 24–72 h.

**Fig. 2:**
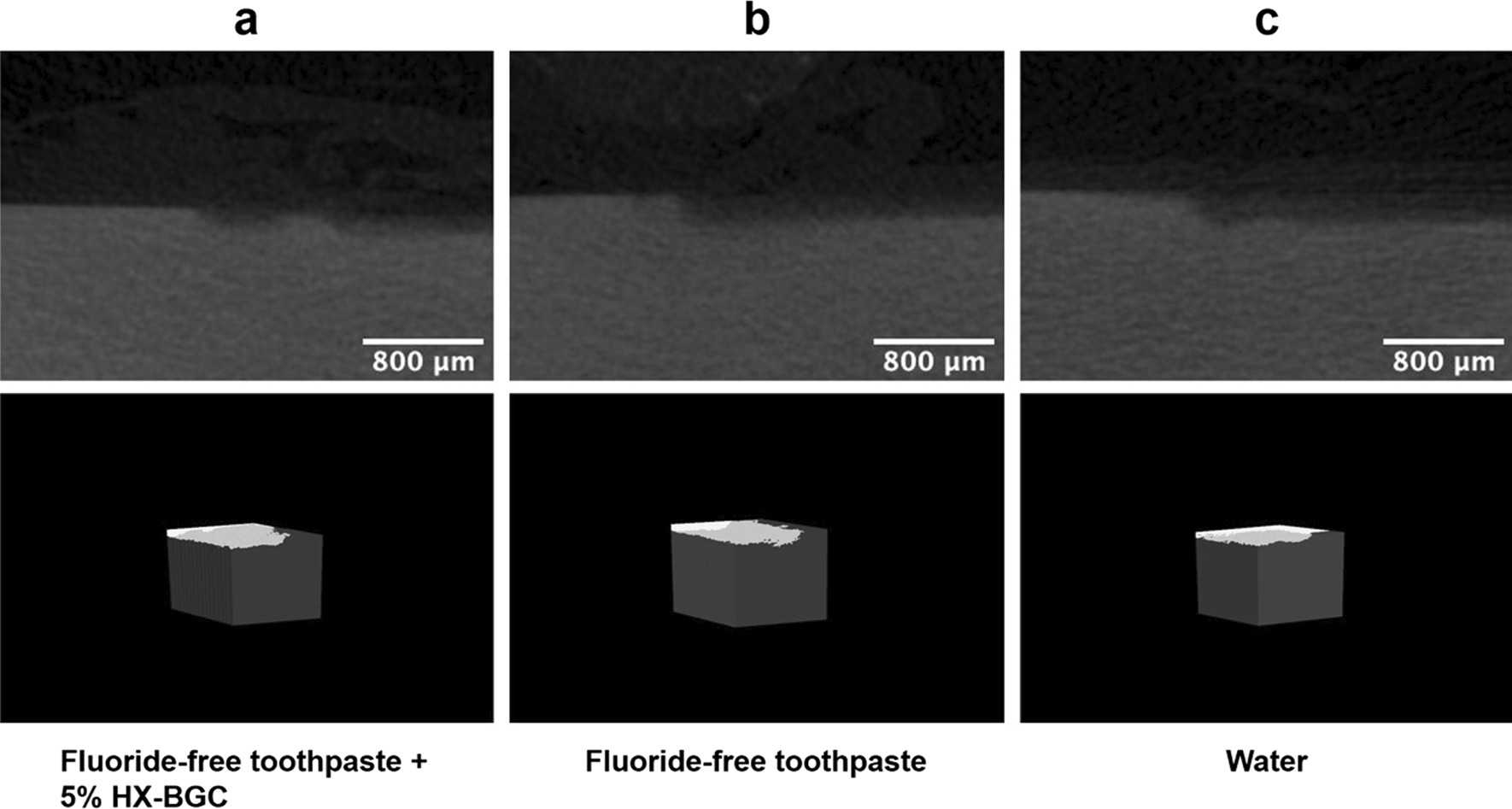
Micro-CT images of the dentine specimens in the three study groups

The mean (± SD) lesion depth in dentine in Group 1 was 87.79 ± 16.99 μm, which was smaller than that in Group 3 (113.60 ± 16.36 μm) (*p* = 0.002). There was no significant difference in mean lesion depth in dentine between Group 2 (101.06 ± 10.04 μm) and Group 3 (*p* = 0.21). There was also no significant difference between Group 1 and Group 2. The mean mineral loss in Groups 1–3 were 0.82 ± 0.10 g/cm^3^, 0.89 ± 0.09 g/cm^3^ and 0.96 ± 0.11 g/cm^3^, respectively. According to the multiple comparison results, the mineral loss in Group 1 was significantly less than that in Group 3 (*p* = 0.013), while there was no significant difference in mineral loss between Group 2 and Group 3 (*p* = 0.394). Details of the mean lesion depths and mineral loss are displayed in Table 1. Figure 2 shows the micro-CT images of specimens in the three study groups. Three dimensional images of artificial caries lesions and non-demineralized dentine were reconstruncted. There were no obvious differences among the three groups (Fig. 2).

**Table 1:** Mean lesion depths and mineral loss of the dentine in three study groups

Groups	Lesion depth (μm)	Mineral loss (g/cm^3^)
Fluoride-free toothpaste + 5% HX-BGC (1)	87.79 ± 16.99	0.82 ± 0.10
Fluoride-free toothpaste (2)	101.06 ± 10.04	0.89 ± 0.09
Water (control) (3)	113.60 ± 16.36	0.96 ± 0.11
Multiple comparison	(1) < (3), (2) = (3)	(1) < (3), (2) = (3)

**Fig. 3:**
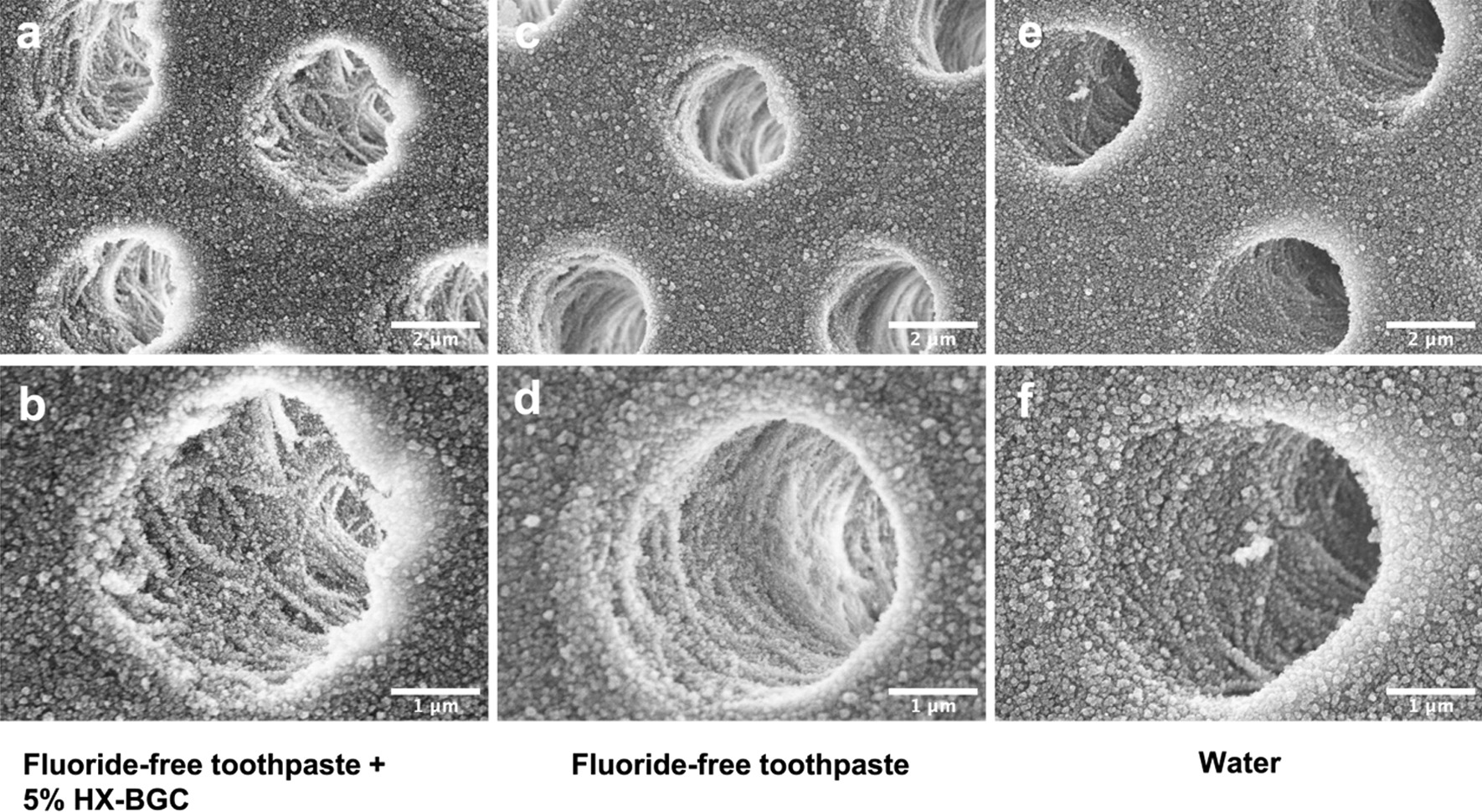
SEM images of transverse dentine surface of the dentine specimens in the three study groups. **a** 10,000 × magnification view of Group 1 (Fluoride-free toothpaste + 5% HX-BGC); **b** 20,000 × magnification view of Group 1; **c** 10,000 × magnification view of Group 2 (Fluoride-free toothpaste); **d** 20,000 × magnification view of Group 2; **e** 10,000 × magnification view of Group 3 (Water); **f** 20,000 × magnification view of Group 3

Figure 3 shows the SEM images of the transverse section of the dentine surface of the specimens in the three study groups. In Group 1, particles were densely integrated together in the inter-tubular area (Fig. 3a), while under higher magnification, the collagen fibers were seen to be relatively intact and obviously exposed in the dentinal tubules (Fig. 3b). Clear dentine structure was observed in Group 2 and in Group 3 without collagen loosening and destruction (Fig. 3c–f). There were no obvious differences in the appearance of the transverse dentine surface among the three groups.

## Discussion

This study investigated the effect of strontium-doped bioactive glass-ceramic containing toothpaste on the formation of artificial dentine caries. The results show that HX-BGC containing toothpaste have the potential to reduce the formation of dentine caries.

An earlier in vitro study found that adding bioactive glass into restorative filling material reduced the extent of biofilm penetration into pre-existing marginal gaps which showed an antibacterial effect of bioactive glass [15]. One of the mechanisms of bioactive glass on caries management is the presence of antibacterial ions which can suppress bacterial growth [16]. Incorporation of strontium into bioactive glass can improve the antimicrobial effect on inhibiting the growth of cariogenic bacteria. The antibacterial effect of strontium has been reported in several studies [17, 18]. A previous study found that the antibacterial ability against *P. gingivalis* depended on the concentration of strontium, while the antimicrobial effect against *A. actinomyces temcomitans* increased only slightly as the strontium concentration increased [18]. Another study found that presence of strontium in the culture medium reduced bacterial cell counts but a concentration of strontium above 14 ppm did not further improve its bactericidal effect [17]. In the present study, the shallower lesion depth and less mineral loss in Group 1 when compare to those in the other two study groups may be attributed to the release of strontium from HX-BGC, which can reduce the acid-producibility of the established biofilm during the demineralization phase of the cycling.

When bioactive glass is exposed to water (such as the artificial saliva in the present study), the pH would increase with dissolution of ions, resulting in precipitation of hydroxyapatite [19]. The lesion center serves as a nucleation place for mineral deposition, which occurs by the regrowth of residual mineral crystals rather than the precipitation of minerals on the organic matrices [20]. A previous study investigated the remineralizing effect of a toothpaste containing a bioactive glass (Bioglass 45S5) on artificially induced carious lesion and found that the study toothpaste increased the level of calcium which could be considered as an effective agent for mineralization [21]. Bioglass 45S5 (45% SiO_2_, 24.5% Na_2_O, 24.5% CaO, 6% P_2_O_5_) paste applied on tooth surface can form a layer rich in calcium phosphate which is resistant to acid and can transform to hydroxyapatite crystals. A study using energy-disperse X-ray analysis showed that the component of the newly formed layer was mainly composed of calcium and phosphorus containing silica which came from the ions of Bioglass 45S5 [22]. The two studies mentioned above have illustrated that bioactive glass can inhibit demineralization by promoting precipitation of hydroxyapatite on tooth surface. In the present study, it is not certain if the minerals observed under SEM in Group 1 were newly precipitated though, the mineral loss in Group 1 was significantly less than that in the control group. It is likely that newly formed apatite had precipitated on the dentine surface of the study specimens. In addition, we only visualized the morphological changes of collagen via SEM in the current study, it would be comprehensive if additional evaluation methods such as FTIR, XRD and XRF were used to prove the changes of collagen.

Autoclaving is an easily accessesible method for sterilization of extracted teeth [23]. Autoclaving may weaken tooth structure but the effect of reduction on microhardness is minimal and does not affect the physical properties [24]. In addition, the molecular structure of dentine collagen can remain intact though autoclaving may destroy the structure slightly [25]. The modified biochemical cycling model used in this study has the advantage of not only causing mineral loss in dentine structure, but also destruction of organic component, which resembles the clinical situation. In this study, only five species of cariogenic bacteria were used to conduct a biofilm challenge for the demineralization process during the cycling. The pH changes of the bacterial suspension at different time points over 72 h were monitored. Then the pH dropped to 4 at 24 h and kept steady during 24 h to 72 h. That is the reason why in the present study the bacterial suspensions were pre-cultured for 8 h and then applied onto the surfaces of tooth specimens. It should be noted that having only several species of bacteria cannot fully mimic the real complex micro-environment in the mouth where there are thousands of microorganisms. In addition, this study had only used four 24 h cycles of demineralization and remineralization with two interventions per day within the 5 day period. In real life, caries formation is a continuous process with multiple long-period exposures to demineralization and remineralization. The study time duration of the present study may not be long enough to simulate the real situation and this limitation is due to the strict regulations on using the study laboratory during the COVID-19 pandemic. Further studies using a longer study period should be conducted to investigate the long term effect of caries prevention with the use of HX-BGC containing toothpaste. The similar images observed under SEM suggest that HX-BGC particles might not completely occlude the surface due to the toothpaste slurry not covering the whole dentine surface. Therefore, no precipitation of bioactive glass particles on the dentine surface was seen in this study.

## Conclusion

Based on the study findings and within the limitations, it is concluded that the study strontium-doped bioactive glass-ceramic has potential to reduce formation of dentine lesions.

## Data Availability

All data generated or analysed during the current study are available in HKU Datahub (https://datahub.hku.hk/). The data of SEM (Fig. 3) was not deposited in Electron Microscopy Data Bank (EMDB).
